# Comment on “Effects of Atrazine on Estrogen Receptor α– and G Protein–Coupled Receptor 30–Mediated Signaling and Proliferation in Cancer Cells and Cancer-Associated Fibroblasts”

**DOI:** 10.1289/ehp.1510927

**Published:** 2016-04-01

**Authors:** Nicolas Chevalier, Rachel Paul-Bellon, Patrick Fénichel

**Affiliations:** 1 Institut National de la Santé et de la Recherche Médicale (INSERM), Nice, France; 2 Université de Nice–Sophia Antipolis, Nice, France; 3 Centre Hospitalier Universitaire de Nice, Nice, France

Albanito et al. recently reported that the herbicide atrazine was able to exert an estrogen-like proliferative activity in various cell models, including ovarian and breast cancer cells and cancer-associated fibroblasts, by inducing the expression of several estrogen target genes without binding to or activating the classical estrogen receptor (ER) α. They also showed that G protein–coupled receptor 30 (GPR30) elicited phosphorylation of extracellular-signal-regulated kinase (ERK) 1/2 and contributed to the proliferative effects of atrazine. We suggest that endocrine disruptors should be carefully examined in different cell models in order to determine the complex mechanistic and functional outcomes that result from the interaction between several receptors and their associated signaling pathways.

Using the JKT-1 cell line derived from a human testicular seminoma (Bouskine et al. 2010) and seminoma tumors, the most frequent testicular germ cell tumors, we reported that seminoma cells expressed ERβ but not ERα (Roger et al. 2005). At physiological concentrations, 17β-estradiol (E_2_) was able to suppress JKT-1 cell proliferation *in vitro* through ERβ (Roger et al. 2005). However, when E_2_ was conjugated to bovine serum albumin, which does not cross the membrane, we observed cell proliferation dependent on ERK1/2 phosphorylation and a nongenomic pathway involving a membrane G protein–coupled estrogen receptor (Bouskine et al. 2008) that we later identified as GPR30 (Chevalier et al. 2012a; Chevalier et al. 2012b).

Similar results were obtained with bisphenol A (BPA) at nanomolar concentrations (Bouskine et al. 2009). In our model, activation of protein kinase A (PKA) was essential for BPA to promote JKT-1 cell proliferation and lead to phosphorylation of cAMP response-element–binding protein (Bouskine et al. 2009). The proliferative effects of BPA on JKT-1 cells did not involve ERK1/2 activation, but rather activation of the protein kinase G (PKG) pathway, which is known to be Gα_i_/Gα_q_-dependent and is involved in BPA activation of calcium influx in pancreatic islet α cells (Alonso-Magdalena et al. 2005). We assumed that these opposite effects of E_2_ and BPA on JKT-1 cell proliferation were linked to the different affinities of BPA for classical and nonclassical estrogen receptors; that is, E_2_, which has a low affinity for GPR30 and a high affinity for ERβ, induced a suppressive effect, whereas BPA, with a low affinity for ERβ and a high affinity for GPR30, induced a promoting effect.

**Figure 1:**
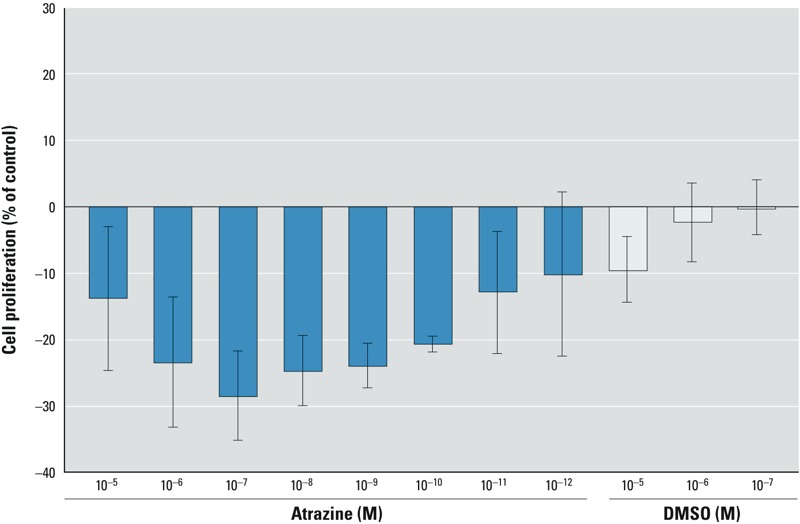
Suppression of JKT-1 cell proliferation by 24-h exposure to various doses of atrazine. Values shown are the percent change in cell number compared with control (steroid-free medium containing DMSO) given as the mean ± SE of three independent experiments. Data from Fénichel et al. (2013).

However, in Fénichel et al. (2013) we also showed in these seminoma cells that atrazine was able to suppress JKT-1 cell proliferation following a linear dose–response curve (Figure 1), contrary to what Albanito et al. reported in other cancer cell lines. This suppressive effect involved the same receptor, as G15 (a selective antagonist of GPR30) was able to reverse the inhibitory effect of atrazine on JKT-1 cell proliferation.

Our results showed that *1*) two chemical compounds (BPA and atrazine) can exert opposite estrogenic effects in the same cancer cell (seminoma) through the same receptor (GPR30), and *2*) the same chemical compound (atrazine) can exert opposite effects in different cancer cells (seminoma, ovarian, or breast) through the same GPR30 receptor. This highlights the role of associated GPR30-induced signaling pathways (ERK1/2, PKA, or PKG) and possible cross-talk between GPR30 and the classical estrogen receptors ERα or ERβ, as suggested by Albanito et al.
